# Obituary

**Published:** 1919-10

**Authors:** 


### OBITUARY

Dr. John F. Dowsley died July 17, 1919, at his home in Newton Center, Mass., a suburb of Boston. Dr. Dowsley was in his sixty-sixth year and died of heart failure. He was one of the best-known dentists of the country and his death will be a great shock to his many friends as well as a severe loss to the profession to which he has been sincerely devoted for thirty-five years. He was constant in his attendance at the meetings of the National Dental Association, his home, and many other local and district societies. He was a most capable and popular practitioner, and served as a member of the Massachusetts State Board of Dental Examiners for twenty-seven years. He was one of the incorporators of the Forsythe Dental Infirmary and an enthusiastic supporter of the hygiene work in Boston and his state. His capacity for work and his intense interest in dentistry made him a most useful member of the profession. His courteous and friendly attitude characterized his leadership of men. He spent his life with rare devotion to the best interests of his patients and the profession and his work will long survive.

